# Ultrasonographic differentiation of diffuse large B-cell lymphoma and mucosa-associated lymphoid tissue lymphoma in primary thyroid lymphoma

**DOI:** 10.3389/fendo.2026.1743975

**Published:** 2026-02-18

**Authors:** Xining He, Ying Bai, Si-fan Wang, Qianyuan Chen, Haiyan Cao, Peipei Xu, Mengyun Yao, Wenjing Zhang, Zhen Wang, Xiuhua Wang, Jiajia Xiong, Feixiang Xiang, Cheng Yu, Jia-wei Shi

**Affiliations:** 1 Department of Ultrasound Medicine, Union Hospital, Tongji Medical College, Huazhong University of Science and Technology, Wuhan, China; 2 Chongqing Hospital, Union Hospital, Tongji Medical College, Huazhong University of Science and Technology, Chongqing, China; 3 Clinical Research Center for Medical Imaging in Hubei Province, Wuhan, China; 4 Hubei Province Key Laboratory of Molecular Imaging, Wuhan, China; 5 Department of Pathology, Union Hospital, Tongji Medical College, Huazhong University of Science and Technology, Wuhan, China

**Keywords:** differential diagnosis, diffuse large B cell lymphoma, mucosa-associated lymphoid tissue lymphoma, primary thyroid lymphoma, ultrasonography

## Abstract

**Purpose:**

The aim of this study was to evaluate the ultrasonographic differences between diffuse large B-cell lymphoma (DLBCL) and mucosa-associated lymphoid tissue (MALT) lymphoma in primary thyroid lymphoma (PTL).

**Methods:**

A total of 46 patients with histopathologically confirmed PTL (27 with DLBCL and 19 with MALT lymphoma) were included in this study. All patients underwent ultrasonographic imaging prior to initiation of therapy. We retrospectively reviewed all images and compared the imaging findings between the two pathologies.

**Results:**

DLBCL was more likely to demonstrate greater clinical aggressiveness than MALT lymphoma, as indicated by a significantly higher rate of perithyroidal tissue invasion (41% vs. 11%, *P* = 0.025) and a lower proportion of asymptomatic cases (19% vs. 47%, *P* = 0.036). The percentage of participants with lesions showing markedly hypoechoic echogenicity on ultrasound was 52% (14 of 27) in DLBCL group, compared with 5% (1 of 19) in MALT group (*P* = 0.001). Additionally, the mean maximum diameter of lesions was 62.5 ± 29.6 mm in DLBCL versus 38.1 ± 21.7 mm in MALT lymphoma (*P* = 0.004). Multivariable analysis showed that only hypoechoic lesions were independently associated with DLBCL (odds ratio 0.08; 95% CI 0.01–0.82).

**Conclusions:**

DLBCL is frequently characterized by markedly hypoechoic echogenicity, larger lesion size, and perithyroidal invasion on ultrasonography, whereas MALT lymphoma commonly presents with asymptomatic clinical manifestations.

## Introduction

Primary thyroid lymphoma (PTL) is a rare malignancy, accounting for 1%-5% of thyroid cancers and approximately 2% of all extranodal lymphomas (1). PTL encompasses several histologic subtypes, among which diffuse large B-cell lymphoma (DLBCL) and extranodal marginal zone lymphoma of mucosa-associated lymphoid tissue (MALT) lymphoma represent the most prevalent forms. MALT lymphoma typically follows an indolent clinical course, whereas DLBCL exhibits aggressive behavior. Importantly, untreated MALT lymphoma can transform into DLBCL, resulting in accelerated disease progression (2). Radiation therapy (RT) is a principal treatment modality for PTL. Single-modality treatment, such as RT alone or surgical excision, is usually reserved for stage IE MALT lymphoma confined to the thyroid gland (3). In contrast, patients with DLBCL typically require multimodal treatment including both immunochemotherapy and radiotherapy (4). Consequently, accurate PTL diagnosis with precise histological subtyping is imperative for therapeutic decision-making and prognostic stratification.

PTL typically manifests as a painless neck mass. Progressive enlargement may cause compressive symptoms including dyspnea, dysphagia, and hoarseness, with symptom profiles varying across histologic subtypes. When clinical and radiological findings suggest PTL, histopathological confirmation via fine-needle aspiration (FNA), core needle biopsy (CNB), or open surgical biopsy is mandatory. Ultrasonography, a noninvasive and widely accessible tool for thyroid evaluation, demonstrates characteristic PTL features such as marked hypoechogenicity, posterior acoustic enhancement, and internal strand-like echoes. These sonographic patterns facilitate discrimination between PTL and other thyroid pathologies (5). Computed tomography (CT) further contributes to staging through assessment of lesion extent, local tissue invasion, and distant metastasis. Notably, Tomohiro Ando et al. (6) reported distinct CT patterns for DLBCL and MALT subtypes, suggesting the potential utility of imaging in histological classification.

As the first-line imaging modality for thyroid disorders, ultrasonography plays an indispensable role in diagnostic workflows and therapeutic planning. To our knowledge, no prior studies have systematically compared sonographic characteristics between DLBCL and MALT subtypes in PTL (6–8). Herein, we present a novel analysis of ultrasound features distinguishing these two histologic variants, with the aim to establish imaging criteria supporting noninvasive PTL subtyping.

## Methods

### Patient selection

**Figure 1:**
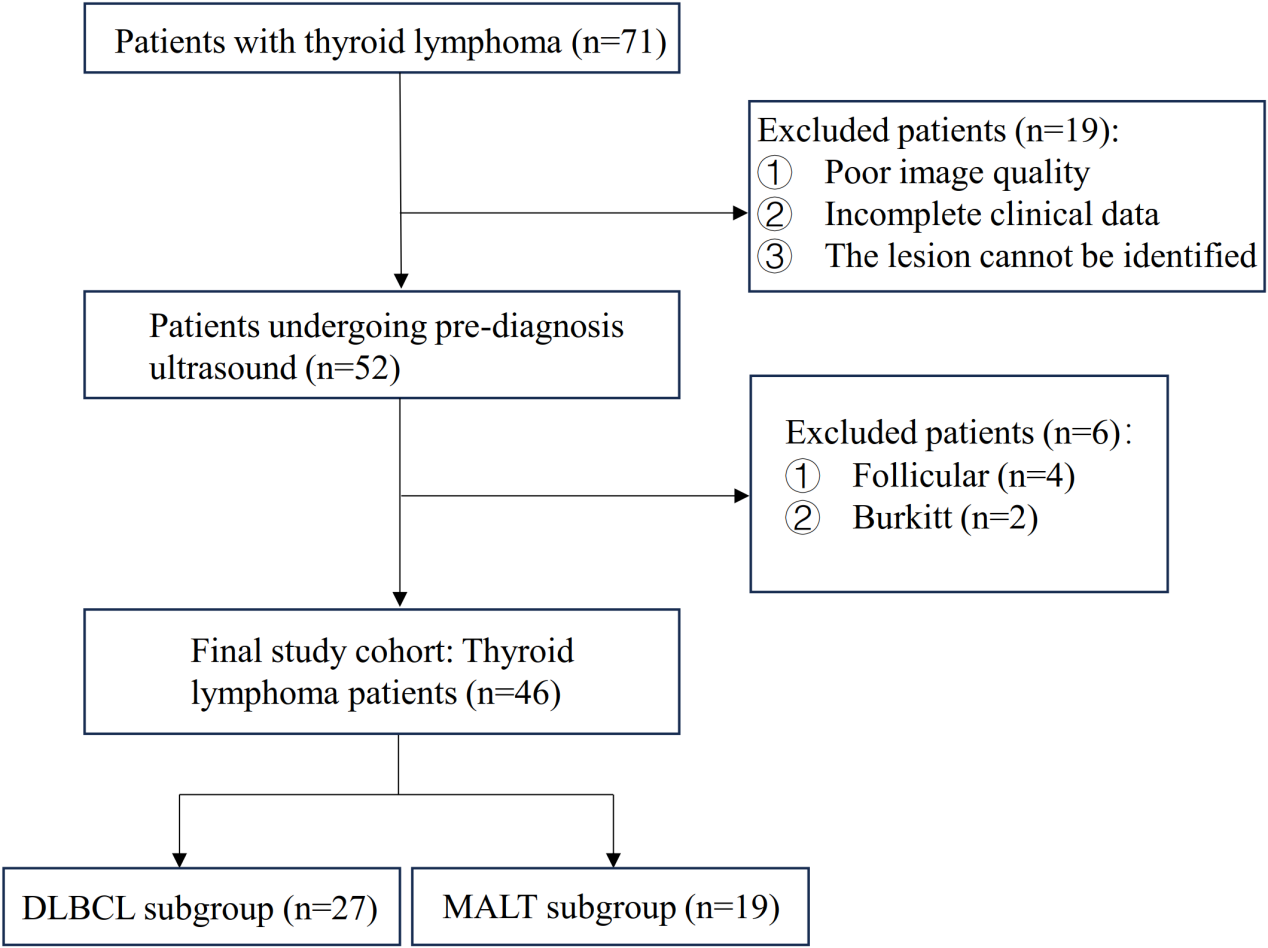
Patient selection flowchart.

Patients with histologically confirmed PTL were identified from the pathological database of our institution between January 2011 and December 2024. Detailed clinical information for these patients was retrieved from the electronic medical record (EMR) system. An initial cohort of 71 patients with PTL was enrolled (**Figure 1**). These cases were further subclassified by immunohistochemical analysis. We excluded patients with poor-quality imaging, those with primary thyroid lymphoma subtypes other than MALT and DLBCL, and those with unidentifiable lesions (**Figure 1**). MALT was found to have a tendency to transform into DLBCL (2); therefore, patients exhibiting coexistence of DLBCL and MALT were classified as DLBCL cases for analysis in this study, totaling five patients. Consequently, 46 patients were included in the final analysis: 27 with DLBCL and 19 with MALT lymphoma (**Figure 1**). Due to the retrospective nature of the study, the requirement for informed consent was waived by the Institutional Review Board.

### Imaging technique and interpretation

The clinical characteristics and sonographic features of the included patients were retrospectively reviewed. Ultrasound images were acquired using the following systems: GE LOGIQ E11, GE LOGIQ E9, Mindray DC80 (Mindray, China), and Philips iU22 (Philips Healthcare, The Netherlands). Sonographic analysis was performed independently by two experienced radiologists (with over 20 years and 15 years of thyroid imaging experience, respectively), blinded to clinical information and pathological diagnoses. In cases of disagreement, consensus was reached through discussion.

Clinical data were collected and recorded from the hospital EMR system. Confirmation of tumor invasion into perithyroidal tissues or cervical lymph node metastasis was based on either postoperative histopathology or whole-body PET-CT findings.

For qualitative assessment, the distribution pattern of thyroid lesions was classified into three categories: diffuse, nodular, or mixed types. The ​​diffuse type​​ was defined as lesions involving both thyroid lobes with ill-defined margins, with or without residual normal thyroid tissue. The ​​nodular type​​ was characterized by a well-defined nodule or mass surrounded by normal thyroid tissue. The ​​mixed type​​ was defined as the presence of multiple ill-defined lesions exhibiting a patchy distribution, also surrounded by normal thyroid tissue. Additionally, the following sonographic features were systematically evaluated: isthmus thickening, lesion composition, echogenicity, margin, shape, presence of calcification, presence of internal fibrous strands, posterior acoustic enhancement, and internal vascularity assessed by color Doppler imaging. Lesion composition was categorized as solid, cystic, or partially cystic. Echogenicity was classified as either hypoechoic or markedly hypoechoic, with the latter defined as having echogenicity lower than that of the anterior strap muscles. Lesion margin was categorized as circumscribed or non-circumscribed. Lesion shape was designated as regular (oval-to-round) or irregular. The presence of calcification, presence of internal fibrous strands, and posterior acoustic enhancement were recorded as present or absent. Internal vascularity was assessed via color Doppler imaging and categorized as absent/minimal or increased (9).

### Statistical analysis

Continuous variables were compared between DLBCL and MALT groups using the Mann-Whitney U test. Categorical variables were compared using the Chi-square test or Fisher’s exact test, as appropriate. All statistical analyses were performed using SPSS software (version 29.0, IBM Corp., Armonk, NY, USA). A two-tailed P-value of < 0.05 was considered statistically significant.

## Results

**Figure 2:**
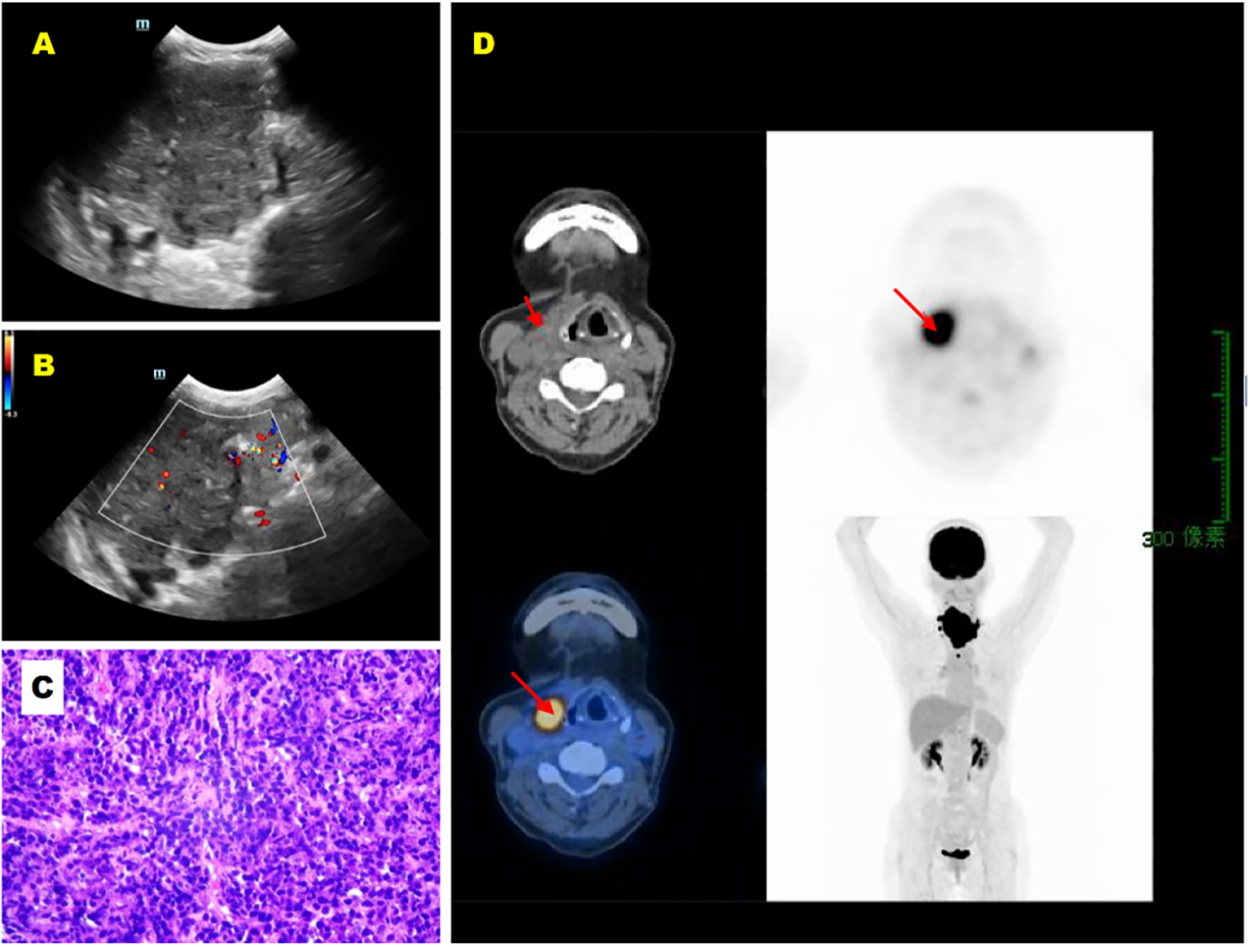
A 57-year-old female patient with primary thyroid DLBCL (diffuse large B-cell lymphoma). **(A)** Longitudinal grayscale ultrasound image of the right thyroid lobe shows an altered gland morphology with significant enlargement, heterogeneous hypoechoic echotexture, and linear hyperechoic stripes. **(B)** Longitudinal color Doppler ultrasound image of the right thyroid lobe demonstrates minimal blood flow signals within the lesion. **(C)** Hematoxylin and eosin (HE) staining of the right thyroid lobe tissue (×200). **(D)** Maximum Intensity Projection image demonstrates a radiotracer-avid focus in the neck. CT reveals a corresponding soft tissue mass with invasion of the thyroid cartilage, showing markedly increased F-luorodeoxyglucose (FDG) uptake. The arrow indicates a right neck soft tissue mass with markedly increased radiotracer uptake.

Patients with DLBCL were generally older than those with MALT lymphoma, although this difference did not reach statistical significance (mean age 63.1 ± 13.1 years vs. 55.7 ± 11.1 years, *P* = 0.052). Both groups exhibited a female predominance. The majority of patients across both lymphoma subtypes had a documented history of Hashimoto’s thyroiditis. DLBCL patients frequently presented with aggressive clinical behavior, including neck mass (89%), tracheal deviation (48%), tracheal stenosis (22%), and dyspnea (26%). Perithyroidal tissue invasion, involving structures such as the trachea, esophagus, larynx, arteries, or thyroid cartilage (**Figure 2**), was observed in 11 DLBCL patients (41%) compared to only 2 MALT lymphoma patients (11%), a difference that was statistically significant (*P* = 0.025). Conversely, a significantly higher proportion of MALT lymphoma patients were asymptomatic (47% vs. 19%, *P* = 0.036). Lymph node involvement was common in both groups (DLBCL: 78%; MALT: 63%), but this difference was not statistically significant (*P* = 0.278). Regarding disease staging, DLBCL was more frequently diagnosed at stage IIE (66%), whereas MALT lymphoma predominantly presented at stage IE(58%). Detailed clinical characteristics, including staging and symptom prevalence, are summarized in **Table 1**.

**Table 1:** Patient characteristics.

Characteristics	DLBCL	MALT lymphoma	*P* value
Number of patients	27	19	/
Age (year)			
Range	35-84	30-74	
Mean ± SD	63.1 ± 13.1	55.7 ± 11.1	0.052
Sex			0.526
Male	10(37%)	6(32%)	
Female	17(63%)	13(68%)	
Clinical stage			0.090
IE	7(26%)	11(58%)	
IIE IV	18(67%) 2(7%)	7(37%) 1(5%)	
Hashimoto’s thyroiditis	23(85%)	17(89%)	1.000
Clinical symptoms			
Neck mass	24(89%)	14(73%)	0.246
Progressive enlargement	16(59%)	8(42%)	0.251
Dysphagia	4(15%)	3(16%)	1.000
Dyspnea	7(26%)	3(16%)	0.488
Hoarseness	2(7%)	0(0)	0.504
Tracheal deviation	13(48%)	4(21%)	0.073
Tracheal stenosis	6(22%)	4(21%)	1.000
Asymptomatic	5(19%)	9(47%)	**0.036**†

DLBCL, diffuse large B cell lymphoma; MALT lymphoma mucosa associated lymphoid tissue lymphoma; SD, standard deviation.

†, *P* < 0.05.

**Figure 3:**
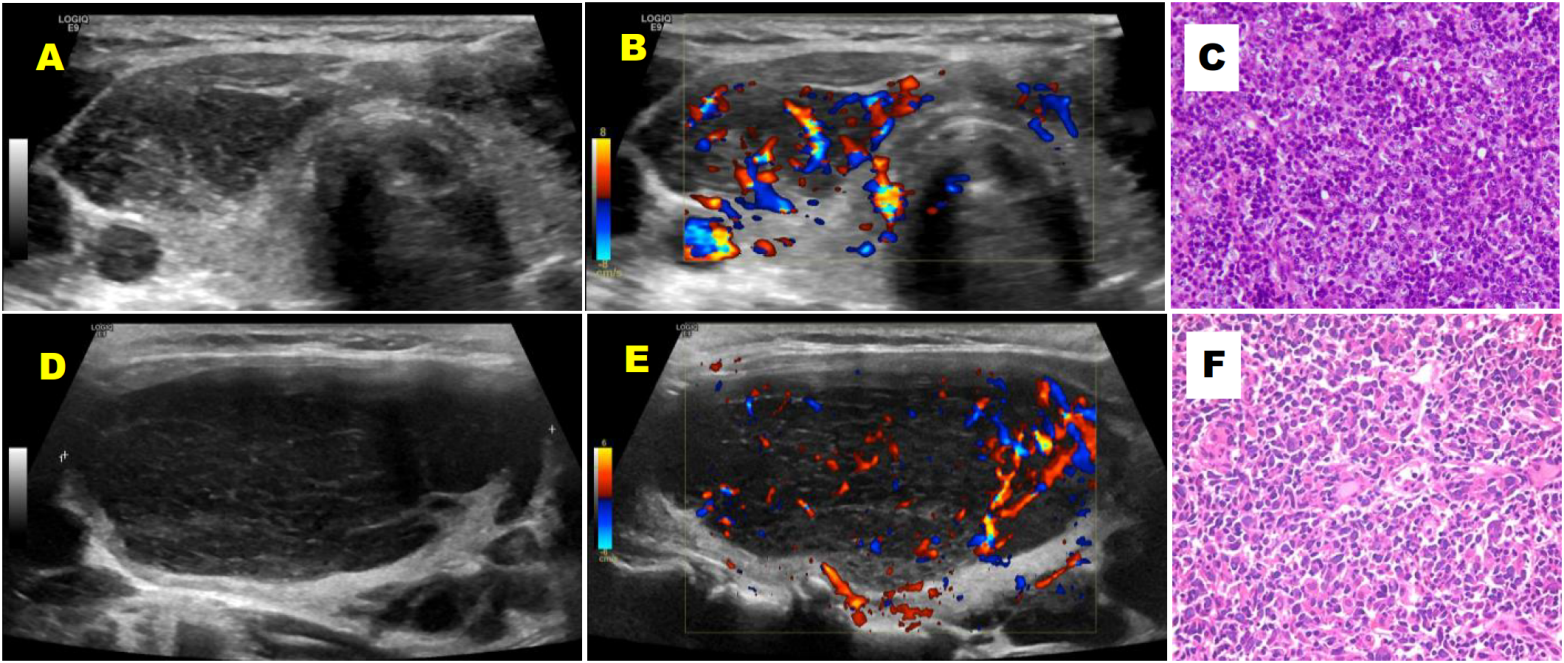
A 43-year-old female patient with primary thyroid MALT lymphoma **(A-C)** and a 59-year-old female patient with primary thyroid DLBCL **(D-F)**. **(A)** Transverse grayscale ultrasound image of the right thyroid lobe shows a hypoechoic area with heterogeneous internal echogenicity and a reticular pattern. **(B)** Transverse color Doppler ultrasound image of the right thyroid lobe reveals rich blood flow signals within the hypoechoic area. **(C)** Hematoxylin and eosin (HE) staining of the right thyroid biopsy tissue (×200). **(D)** Longitudinal grayscale ultrasound image of the left thyroid lobe shows a full gland with rounded contours, containing a large, markedly hypoechoic area with heterogeneous internal echogenicity and linear hyperechoic stripes. **(E)** Longitudinal color Doppler ultrasound image of the left thyroid lobe reveals rich blood flow signals within the markedly hypoechoic area. **(F)** Hematoxylin and eosin (HE) staining of the biopsy tissue from the left thyroid mass (×400).

Both DLBCL and MALT lymphoma most commonly exhibited a nodular growth pattern. Some lesions demonstrated a diffuse distribution, while a mixed pattern was observed less frequently. There was no statistically significant difference in the distribution patterns between the two subtypes. The distribution of lymphoma lesions demonstrated notable trends: MALT lymphoma lesions occurred more frequently in the bilateral thyroid lobes (58%), while DLBCL lesions predominantly involved unilateral thyroid involvement (63%). However, this observed difference did not reach statistical significance (*P* = 0.162). In contrast, a highly significant difference was observed in lesion echogenicity. Among the 27 patients with DLBCL, 14 (52%) had lesions that were predominantly markedly hypoechoic, as compared with 1 of 19 patients (5%) with MALT lymphoma (*P* = 0.001, **Figure 3**, **Table 2**). The maximum lesion diameter was significantly greater in DLBCL compared to MALT lymphoma (62.5 ± 29.6 mm vs. 38.1 ± 21.7 mm, *P* = 0.004, **Figures 3A, D**, **Table 2**). The DLBCL and MALT groups showed similar findings in terms of calcifications (7% vs. 5%), internal fibrous strands (63% vs. 68%), posterior acoustic enhancement (15% vs. 11%), and rich vascularity assessed by color Doppler flow (70% and 74%; **Table 2**, **Figure 3**).

**Table 2:** Qualitative assessments and quantitative measurements.

	DLBCL (n=27)	MALT lymphoma (n=19)	*P* value
Bilateral Morphological pattern	10(37%)	11(58%)	0.162 0.608
Diffuse	11(41%)	6(32%)	
Nodular Mixed	13(48%) 3(11%)	9(47%) 4(21%)	
Lymph node metastasis	21(78%)	12(63%)	0.278
Invasion of surrounding tissues	11(41%)	2(11%)	0.025†
Preserved peripheral thyroid tissue	17(63%)	12(63%)	0.989
Thickening of the isthmus Margin Circumscribed Uncircumscribed Shape Regular Irregular Echogenicity Hypoechoic Markedly hypoechoic Echogenic strands Calcification	17(63%) 9(33%) 18(67%) 3(33%) 6(67%) 13(48%) 14(52%) 17(63%) 2(7%)	12(63%) 10(53%) 9(47%) 6(60%) 4(40%) 18(95%) 1(5%) 13(68%) 1(5%)	0.989 0.191 0.370 0.001† 0.702 0.772
Posterior echo enhancement	4(15%)	2(11%)	1.000
Maximum diameter (mm)	62.5 ± 29.6‡	38.1 ± 21.7‡	0.004†
Vascularity Absence/not rich vascularity Rich vascularity	8(30) 19(70)	5(26) 14(74)	0.806

†, *P* < 0.05.

‡, Mean ± standard deviation.

DLBCL, diffuse large B cell lymphoma; MALT lymphoma, mucosa-associated lymphoid tissue lymphoma.

**Figure 4:**
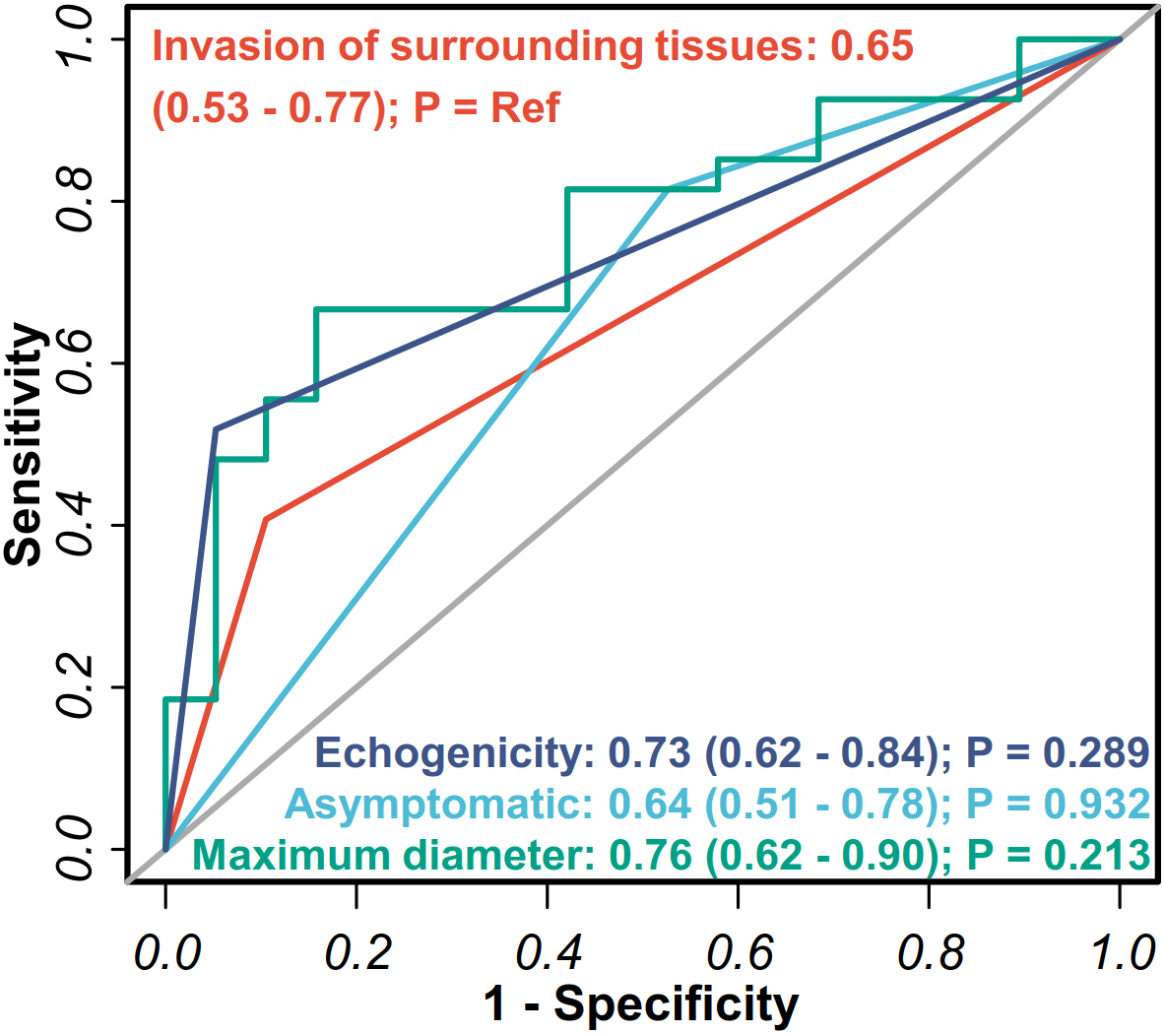
Performance of ultrasonographic and clinical features for the detection of DLBCL and MALT lymphoma in PTL. The analysis evaluated four key parameters: invasion of surrounding tissues, maximum lesion diameter, lesion echogenicity, and asymptomatic presentation. The diagonal gray line represents the reference line of no discriminative ability. DeLong’s test was used to compare the ROC curves, evaluating whether the other three indicators differed significantly from invasion of surrounding tissues.

An assessment of the ability of key features to discriminate DLBCL from MALT lymphoma was conducted using receiver operating characteristic (ROC) curves. The maximum lesion diameter showed the highest diagnostic performance with an AUC of 0.76 (95% confidence interval [CI], 0.62 to 0.90), followed by lesion echogenicity, with AUC of 0.73 (95% CI, 0.62 to 0.84). Perithyroidal tissue invasion (AUC 0.65) and asymptomatic presentation (AUC 0.64) demonstrated moderate discriminatory value. However, DeLong’s test showed that none of the differences in AUC among these features were statistically significant (all *P* > 0.05; **Figure 4**).

Logistic regression analysis was undertaken to identify significant predictors for differentiating DLBCL from MALT lymphoma. Four variables were significantly associated with a diagnosis of DLBCL, including the presence of clinical symptoms (OR, 3.96; *P* = 0.042), larger maximum lesion diameter​ (OR, 1.04; *P* = 0.008), hypoechoic echogenicity (OR, 0.05; *P* = 0.007), and invasion of surrounding tissues (OR, 5.84; *P* = 0.036) (**Table 3**). In the multivariable logistic regression analysis controlling for patient age and sex, only markedly hypoechoic echogenicity​ remained an independent and strong predictor of DLBCL (Adjusted OR=13.12, 95% CI: 1.22-140.89, *P* = 0.034) (**Table 3**). A comparative analysis was subsequently performed regarding the application of preoperative pathological examinations in the two groups. Among the 46 patients with PTL, 19 underwent preoperative FNA or CNB, including 12 patients (44%) in the DLBCL group and 7 patients (37%) in the MALT group (*P* = 0.606; **Table 4**). Of these, 11 patients had FNA, and none received an accurate pathological subtype; 6 cases were misdiagnosed as Hashimoto’s thyroiditis. The diagnostic yield of FNA did not differ significantly between the two groups (5 of 7 in DLBCL vs. 1 of 3 in MALT, *P* = 0.197; **Table 4**). All 8 patients who underwent CNB were diagnosed with PTL, and 7 of them were accurately subtyped (*P* = 0.625; **Table 4**).

**Table 3:** Multivariable predictors of differentiating DLBCL from MALT lymphoma in PTL.

Characteristic	Univariable		Multivariable	
	Odds ratio (95%CI)	*P*	Odds ratio (95%CI)	*P*
Age	1.05 (1.00-1.11)	0.06	1.03 (0.96-1.09)	0.434
Sex: Female	0.78 (0.23-2.72)	0.702	3.33 (0.49-22.69)	0.220
Asymptomatic: No	3.96 (1.05-14.89)	0.042	1.88 (0.32-10.94)	0.480
Maximum diameter	1.04 (1.01-1.07)	0.008†	1.02 (0.99-1.06)	0.228
Echogenicity: Markedly Hypoechoic	19.38 (2.26-166.50)	0.007†	13.12 (1.22-140.89)	0.034†
Invasion of surrounding tissues: Yes	5.84 (1.12-30.55)	0.036†	2.80 (0.38-20.92)	0.315

†, *P* < 0.05.

**Table 4:** Diagnostic workup by primary thyroid lymphoma subtype.

	Overall (n=46)	DLBCL (n=27)	MALT lymphoma (n=19)	*P*
Pre-operative diagnosis FNA	19(41%) 11	12(44%) 7	7(37%) 4	0.606 0.197‡
Hashimoto’s thyroiditisNHL/cancer CNB Cancer† DLBCL/MALT	6(55%)5(45%) 8 1(13%) 7(88%)	5(71%)2(29%) 5 1(20%) 4(80%)	1(25%)3(75%) 3 0(0) 3(100%)	0.625‡

CNB, Core Needle Biopsy; DLBCL, diffuse large B cell lymphoma; FNA, Fine-Needle Aspiration; MALT lymphoma, mucosa-associated lymphoid tissue lymphoma; NHL, non-Hodgkin lymphoma.

†Indicates that preoperative cytology or histology confirmed malignancy, but the specific lymphoma subtype (e.g., DLBCL or MALT) could not be determined.

‡Fisher’s exact test was performed.

## Discussion

Precise discrimination between DLBCL and MALT lymphoma is clinically imperative due to their divergent prognostic trajectories and therapeutic requirements. The present study identified statistically significant differences between DLBCL and MALT lymphoma in terms of maximum lesion diameter, echogenicity pattern, prevalence of local invasion, and frequency of asymptomatic presentation. These parameters may aid in the preoperative differential diagnosis of PTL subtypes and potentially allow selected patients to avoid diagnostic surgery.

The study cohort demonstrated demographics characteristic consistent with existing literature (10), featuring predominantly female patients (mean age >50 years) with frequent background Hashimoto’s thyroiditis. Chronic antigenic stimulation likely drives the gradual lymphomagenesis observed in this context (11). Clinically, patients typically presented with rapidly enlarging cervical masses inducing compressive symptoms, with both subtypes exhibiting substantial lesion. Notably, DLBCL lesions had significantly larger maximal diameters (62.5 ± 29.6 mm vs. 38.1 ± 21.7 mm; *P* = 0.004), occasionally involving entire thyroid lobes. The aggressive nature of DLBCL manifested through frequent local invasion (41% vs. 11%; P = 0.025) and higher lymph node involvement (78%), with clinical presentations including palpable neck masses (89%), tracheal deviation (48%), tracheal stenosis (22%), and dyspnea (26%). Conversely, MALT lymphoma exhibited indolent behavior with greater asymptomatic prevalence (47% vs. 19%; P = 0.036) and superior 5-year survival rates (90-96%) (12). These divergent clinical profiles underscore the necessity for rapid subtype differentiation, as localized MALT lymphoma may be managed with surgery alone, whereas DLBCL requires multimodal therapy.

While histopathology remains the diagnostic gold standard, advanced imaging modalities contribute significantly to staging and characterization. Computed tomography provides comprehensive assessment from head to pelvis. Tomohiro Ando et al. (6) have identified several discriminative CT features, including lesion size, morphological patterns, isthmus thickening, local invasion, and residual thyroid parenchyma. Fluorodeoxyglucose positron emission tomography (FDG-PET) further aids in monitoring treatment response and detecting recurrence (2, 13). Within this diagnostic framework, ultrasonography provides unique value through its ability to detect subtype-specific patterns. DLBCL lesions predominantly demonstrated markedly hypoechoic echogenicity (52% vs. 5%; *P* = 0.001), corresponding histopathologically to their destructive architecture with diffuse sheets of atypical lymphocytes, necrosis, and “starry-sky” macrophages. In contrast, the hypoechoic appearance of MALT lymphoma reflected marginal zone infiltration and lymphoepithelial lesions. Both subtypes exhibited rich vascularity (DLBCL: 70%; MALT: 74%) and shared sonographic features such as posterior enhancement and internal strands, likely due to homogeneous tumor cellularity and perilesional fibrosis reactions.

Distribution patterns (diffuse, nodular, mixed) presented diagnostic challenges, particularly in diffuse-type PTL where sonographic similarities to Hashimoto’s thyroiditis necessitate vigilance for rapid enlargement and abnormal nodal architecture. These observations align with cross-organ lymphoma studies documenting consistent sonographic patterns of hypoechogenicity and hypervascularity (14–17). While our findings establish clinically valuable differentiators for preliminary subtype assessment, ultrasound’s inherent limitations necessitate histological confirmation via fine-needle aspiration, core needle biopsy, or surgical sampling (18). In this study, none of the 11 patients who underwent preoperative FNA received an accurate histological classification. Among these cases, 6 were misdiagnosed as Hashimoto’s thyroiditis (5 DLBCL and 1 MALT). In contrast, only 1 of the 8 patients who underwent preoperative CNB failed to receive a definitive histological subtype. Patients with DLBCL were more likely to undergo preoperative biopsy, likely due to their more pronounced clinical symptoms, which created a greater urgency for establishing a definitive diagnosis to guide clinical management. Diagnosing lymphoma based solely on FNA poses considerable challenges for pathologists. However, incorporating ancillary techniques such as immunohistochemistry (IHC) and flow cytometry can significantly improve diagnostic accuracy. Previous studies (19) have indicated that FNA combined with cell block IHC holds potential for complete subclassification of DLBCL. For patients who are not candidates for surgical intervention, a definitive preoperative diagnosis can help avoid unnecessary surgical trauma and facilitate conservative treatment strategies. Although FNA is less invasive than CNB, it remains limited in its ability to reliably classify lymphoma subtypes (20, 21). A recent meta-analysis reported a substantially higher sensitivity of 94.3% for CNB in diagnosing PTL (22). Furthermore, compared to FNA, CNB reduces the need for diagnostic surgery by more than 20% (18). Accordingly, when PTL is suspected, some authors recommend CNB as the preferred initial diagnostic modality to minimize the burden of additional testing and interventions for patients (23). Nevertheless, pre-biopsy sonographic characterization guides targeted tissue acquisition, enhances sampling adequacy, and contributes to staging through evaluation of perithyroidal tissue involvement.

A major clinical implication of our findings is the role of ultrasound in guiding the diagnostic pathway. Although histopathological confirmation remains mandatory, ultrasound-based estimation of subtype probability may directly impact preoperative management. For patients with markedly hypoechoic, bulky lesions suspicious for DLBCL, clinicians should bypass FNA in favor of core needle biopsy (CNB) to improve diagnostic accuracy and expedite treatment planning. Our data reinforce this approach: FNA demonstrated a high rate of nondiagnostic or misleading results (particularly misclassification as Hashimoto’s thyroiditis), whereas CNB showed substantially higher diagnostic concordance for both DLBCL and MALT. In addition to guiding biopsy selection and early multidisciplinary evaluation, preoperative sonographic differentiation also influences the subsequent decision of whether PET-CT should be incorporated into the diagnostic pathway, particularly in elderly patients (24). PET-CT plays different roles in the two PTL subtypes. When ultrasonographic features strongly suggest DLBCL, PET-CT becomes an important next step, as DLBCL typically demonstrates high FDG avidity and frequently exhibits extranodal or distant involvement (7, 13). Conversely, in lesions with imaging features favoring an indolent subtype such as MALT lymphoma, the necessity of PET-CT is more selective.

For MALT lymphoma, particularly in patients without obvious clinical symptoms, a key challenge lies in differentiating it from other common thyroid diseases. In our cohort, MALT lesions predominantly manifested as hypoechoic areas with relatively homogeneous or finely heterogeneous internal echotexture and a characteristic reticular or mesh-like pattern, often accompanied by increased vascularity on color Doppler imaging. While these features may overlap with chronic Hashimoto’s thyroiditis, distinct warning signs exist. MALT lymphoma should be strongly suspected when a patient with long-standing Hashimoto’s thyroiditis presents with a new focal or asymmetric hypoechoic lesion, shows progressive unilateral or lobar enlargement, or exhibits associated suspicious cervical lymph nodes.

### Study limitations

This retrospective single-center study may be subject to selection bias and the sample size, although collected over 13 years, remains limited due to the rarity of PTL. Despite this limitation, our cohort of 46 patients provides a more comprehensive assessment of clinical and sonographic characteristics than many prior investigations.

## Conclusions

Ultrasonographically, DLBCL more frequently demonstrates markedly hypoechoic echogenicity, larger maximal lesion diameter, and a higher rate of perithyroidal tissue invasion. In contrast, MALT lymphoma is typically characterized by an asymptomatic clinical presentation. These distinct differences provide valuable clues for distinguishing DLBCL from MALT lymphoma in clinical practice.

## Data Availability

The raw data supporting the conclusions of this article will be made available by the authors, without undue reservation.
