# Supplementary figures and images for: Ultrasonographic differentiation of diffuse large B-cell lymphoma and mucosa-associated lymphoid tissue lymphoma in primary thyroid lymphoma
Source: Front Endocrinol (Lausanne). 2026 Feb 18;17:1743975. doi: 10.3389/fendo.2026.1743975 (PMC12957263; doi:10.3389/fendo.2026.1743975)

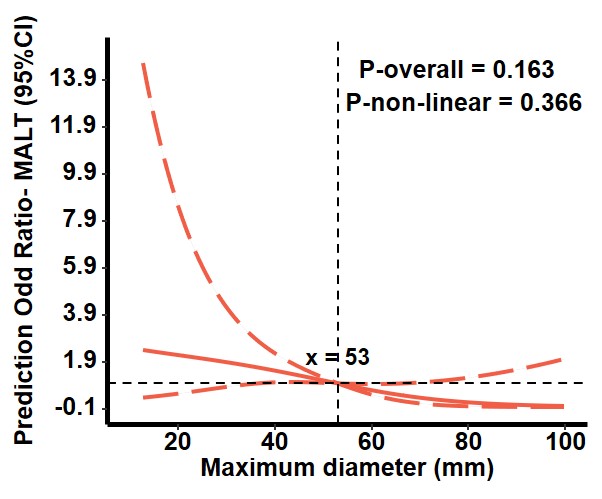

Supplement: Supplementary file 1 [file Image1.jpeg]
